# Prognostic value and determinants of a hypointense core in T_2_-weighted cardiac magnetic resonance in acute reperfused ST-elevation myocardial infarction

**DOI:** 10.1186/1532-429X-13-S1-O85

**Published:** 2011-02-02

**Authors:** Holger Thiele, Ingo Eitel, Konrad Kubusch, Steffen Desch, Oliver Strohm, Yoko Mikami, Suzanne de Waha, Matthias Gutberlet, Gerhard Schuler, Matthias Friedrich

**Affiliations:** 1 University of Leipzig - Heart Center, Leipzig, Germany; 2 Stephenson Cardiovascular Magnetic Resonance Centre at the Libin Cardiovascular Institute of Alberta, Calgary, AB, Canada

## Introduction

A hypointense core of infarcted myocardium in T2-weighted CMR has been used as a noninvasive marker for intramyocardial hemorrhage and was related with adverse remodelling in recently published clinical trials. However, the clinical significance of such findings is not yet established.

## Purpose

Aim of this study was to evaluate determinants and prognostic impact of a hypointense infarct core in T2-weighted cardiovascular MR (CMR) images, studied in patients after acute, reperfused ST-elevation myocardial infarction (STEMI).

## Methods

We analyzed 346 STEMI patients undergoing primary angioplasty <12 hours after symptoms onset at 2 institutions in Germany and Canada. T2-weighted and contrast-enhanced CMR was used for assessment of the area-at-risk, myocardial salvage, infarct size, hypointense core in T2-weighted images and late microvascular obstruction (MO). Patients were categorized into 3 groups defined by the presence or absence of a hypointense core and also MO. Primary endpoint of the study was occurrence of major adverse cardiovascular events (MACE) defined as death, reinfarction and congestive heart failure requiring hospital admission within 6 months after infarction.

## Results

There were 3 groups of patients; patients with hypointense core plus MO (n=122), patients without hypointense core with MO (n=108), and patients without hypointense core and without MO (n=116). The extent of infarct size (r=0.61) and late MO (r=0.74) correlated significantly with the volumetric extent of the hypointense core (p<0.001, respectively). In a multivariable regression model adjusted for significant variables in univariable regression analysis, the extent of late MO (p<0.001), infarct size (p=0.01), and impaired ejection fraction (p=0.02) were the strongest predictors of hypointense cores.

**Figure 1:**
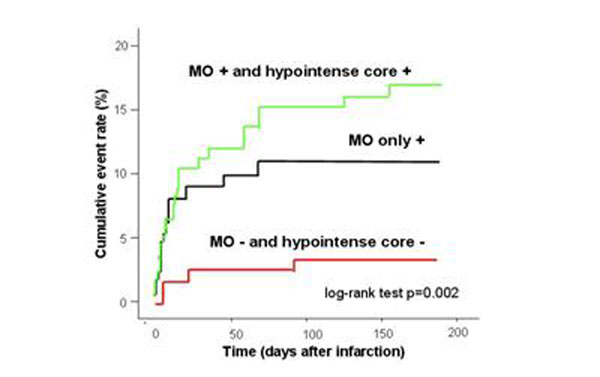


The presence of a hypointense core was a strong univariable and multivariable predictor of MACE (hazard ratio: 2.59, confidence interval: 1.27-5.27). When using a 3-level categorical variable including 1) MO and hypointense core both present 2) MO only present; 3) No MO and no hypointense core present, a risk gradient across the 3 groups could be observed (16.4% versus 10.8% versus 3.6%, p=0.002; Figure 1).

## Conclusions

A hypointense infarct core within the area at risk of reperfused infarcted myocardium in T2-weighted CMR is closely related to infarct size, MO and impaired left ventricular function with subsequent adverse clinical outcome.

